# A Reconfigurable Electro-Optic Photonic Integrated Circuit Architecture

**DOI:** 10.3390/mi17091035

**Published:** 2026-08-29

**Authors:** Paulo Lourenço, André Moreira, Ernesto Velázquez, Alessandro Fantoni

**Affiliations:** 1 Instituto Superior de Engenharia de Lisboa (ISEL), Polytechnic University of Lisbon, 1549-020 Lisboa, Portugal; a47125@alunos.isel.pt (A.M.); a50267@alunos.isel.pt (E.V.); alessandro.fantoni@isel.pt (A.F.); 2 Center of Technology and Systems (CTS) and Associated Lab of Intelligent Systems (LASI), 2829-516 Caparica, Portugal

**Keywords:** reconfigurable photonics, photonic integrated circuits, optical switching, electro-optic modulation, amorphous silicon, multimode interference couplers

## Abstract

In this work, we present the design and numerical validation of a reconfigurable electro-optic photonic integrated circuit architecture for dynamic 1 × 4 optical routing, based on electro-optic phase shifters and a chain of cascaded multimode interference structures, and implemented in an amorphous silicon platform. By exploiting recent developments in symmetry-engineered silicon photonics to enable phase control in a platform compatible with complementary metal-oxide-semiconductor fabrication requirements, this approach addresses the increasing demand for ultra-fast, compact and energy efficient reconfigurable photonic integrated circuits. The photonic circuit design bases its functionality on self-imaging theory and has been optimized through simulations implementing the beam propagation method, while optical performance and electro-optical behavior have been validated through finite difference time-domain simulations and multiphysics modeling. The numerical results obtained confirm the operational performance of the individual building blocks and demonstrate the proposed architecture as being able to perform as a reconfigurable electro-optic platform and provide the dynamic 1 × 4 optical routing. Hence, this architecture provides a scalable platform for reconfigurable silicon photonics, complementary metal-oxide-semiconductor compatible, and the fabrication-ready layout establishes a practical path to experimental validation and future reconfigurable photonic integrated circuits.

## 1. Introduction

The proliferation of data-centric applications such as AI-based instances, cloud computing, optical communications, quantum technologies, and integrated sensing has driven an increasing demand for photonic integrated circuits (PICs). These devices deliver high-speed signal processing while consuming less power, occupying less space, and offering better scalability. By combining various optical functions on a single chip, PICs serve as a vital technological enabler for next-generation photonic systems, providing notable improvements in performance, reliability, and compliance with existing fabrication methods when compared with traditional optical components. As fabrication methods improve and become more compatible with complementary metal-oxide-semiconductor (CMOS) technology, integrated photonics are quickly expanding beyond telecommunications into diverse scientific and industrial fields [1,2,3].

Optical switches are vital components of modern PICs, enabling network reconfiguration, dynamic signal routing, and programmable functionality. Their performance directly affects the energy efficiency, scalability, and speed of optical communication systems, interconnects, emerging optical computing, and sensors [4]. Over the past decade, various switching methods—thermo-optic, micro-electromechanical systems, carrier injection or depletion, and electro-optic—have been explored, each with different trade-offs in loss, power, speed, and fabrication complexity [5,6]. However, achieving compact size, low-loss, ultra-fast operation, power efficiency, and CMOS compatibility simultaneously remains challenging. This drives ongoing efforts and is an excellent research stimulus to develop new photonic switching architectures.

Among the different optical switching architectures developed to overcome these challenges, multimode interference (MMI) devices stand out as especially promising photonic components for programmable and reconfigurable photonic integrated circuits. They operate on the self-imaging principle [7], where the constructive interference of guided modes periodically replicates the input optical field within the multimode interference section. This distinctive propagation behavior enables compact and broadband optical components with relaxed fabrication requirements, low excess loss, and excellent splitting power uniformity [8,9]. As a result, MMI couplers are widely used in combiners, optical routers, integrated splitters, and switching networks. These networks often cascade several interference stages to obtain complex routing functions while maintaining a small footprint and CMOS fabrication compatibility [10,11]. These characteristics make MMI-based architecture especially adequate for reconfigurable, scalable photonic integrated circuits.

MMI couplers, despite their many benefits, are passive optical components that require an external phase control system for dynamic and programmable operation. Consequently, the performance of reconfigurable photonic integrated circuits depends not only on the passive routing architecture but also on the power consumption, speed, and efficiency of the phase shifters used. Thermo-optic phase shifters are more commonly found in modern architectures due to their wide tuning range and ease of implementation; however, their slow response and requirement for uninterrupted power limit their use in high-speed and energy-efficient systems. Faster methods based on free-carrier plasma dispersion are available, but they often increase optical loss and design complexity. These drawbacks have sparked greater interest in electro-optic modulation techniques that can offer low-power, high-speed, and compact phase control while being compatible with integrated photonic platforms [5,6].

Recent progress in symmetry-engineered silicon photonic platforms has demonstrated that effective second-order nonlinearities can be generated in centrosymmetric materials through interface engineering and externally applied electric fields, allowing for electro-optic phase modulation in CMOS-compatible technologies [12,13]. These advancements open new possibilities for energy-efficient, high-speed, compact reconfigurable photonic circuits that remain compatible with silicon foundries. In this work, we present the design and numerical validation of a reconfigurable electro-optic photonic integrated circuit architecture for dynamic 1 × 4 optical routing. This architecture uses cascaded MMI couplers and electro-optic phase shifters on an amorphous silicon platform. We analyze the design through detailed numerical simulations, including beam propagation, finite-difference time-domain, and multiphysics models, demonstrating optical routing between four output ports and laying a practical groundwork for future experimental development.

## 2. Materials and Methods

### 2.1. Device Architecture

The proposed Photonic Integrated Circuit (PIC) was designed using an amorphous silicon (a-Si) platform on a silicon-on-insulator (SOI) substrate. The materials stack, from top to bottom, consists of the following components: an upper gold electrode deposited on silicon dioxide, upper cladding, the optical waveguide core, silicon dioxide lower cladding, a ground electrode, a buried oxide layer, and a crystalline silicon substrate.

**Figure 1:**
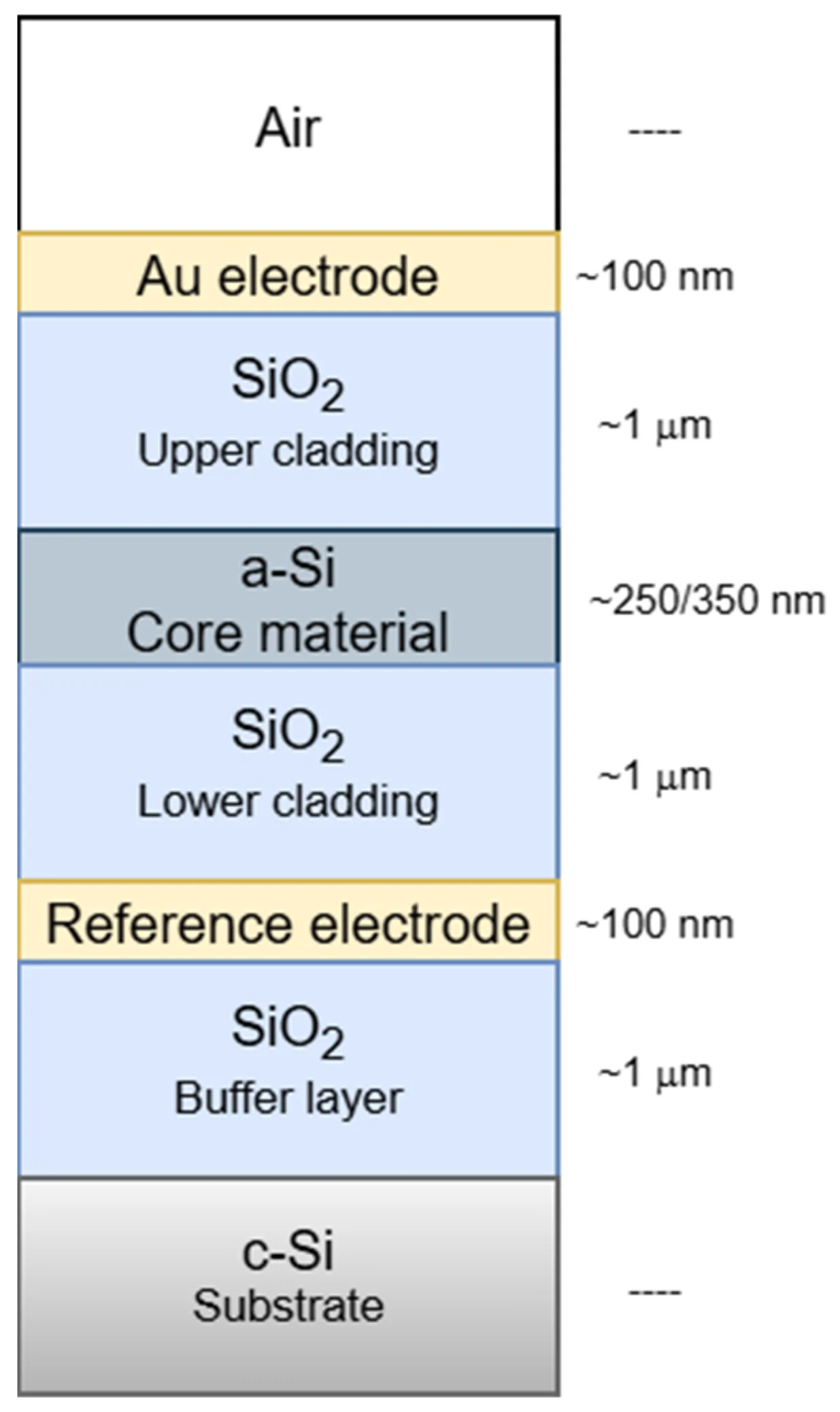
The material stack and approximate thicknesses used for fabrication of the proposed device.

The waveguiding core is made of a 250 nm thick layer of a-Si throughout the circuit. The only exception occurs in the electro-optic racetrack waveguide sections, where an additional 100 nm layer of silicon rich nitride (SiN) is deposited on top of the a-Si core, resulting in a total core height of 350 nm. This hybrid a-Si/SiN configuration was specifically adopted to promote symmetry breaking at the material interface, enabling an effective second-order nonlinear susceptibility ($\chi_{eff}^{2}$) under an externally applied direct current (DC) electric field. As a result, the otherwise centrosymmetric a-Si waveguide exhibits an effective Pockels response, providing the electro-optic phase modulation required by the proposed switching architecture. A detailed description of the adopted electro-optic model is presented in Section 2.4. The complete material stack, as agreed with the fabrication facility, is shown in Figure 1.

**Figure 2:**
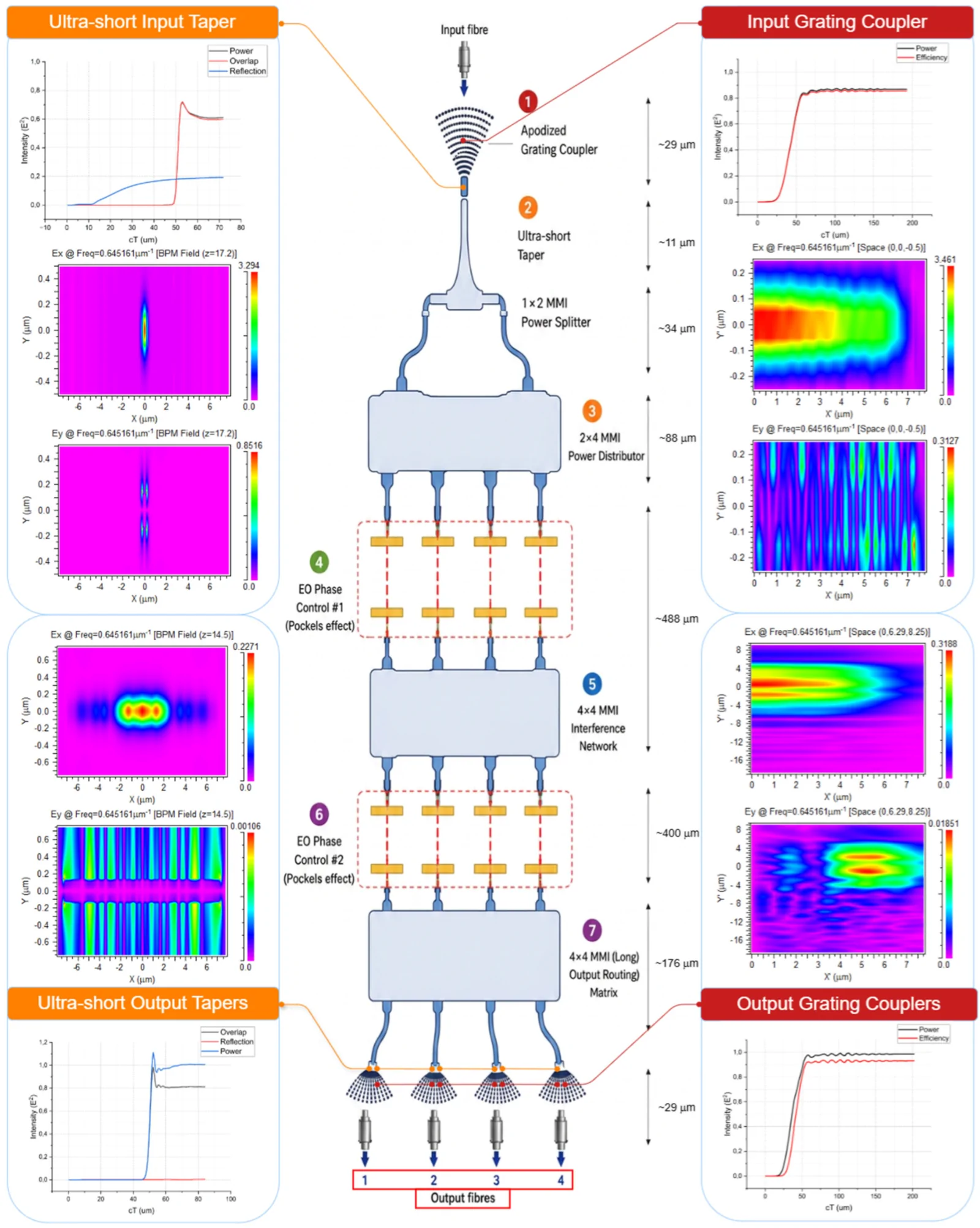
Device’s diagram and highlighted simulation results obtained in both propagation directions: fiber/grating coupler/taper waveguide and taper waveguide/grating coupler/fiber.

This PIC has been designed to operate at a wavelength of 1550 nm using the fundamental Transverse Electric (TE_0_) mode. In this mode, the transverse electric and magnetic field components are aligned along the X- and Y-axes, represented as Ex and Hy, respectively. The development of the integrated optical switch follows a hierarchical design methodology that assembles individually optimized photonic components. All passive and optically active elements—including grating couplers, planar taper waveguides, electro-optic phase-shift regions, and Multimode Interference (MMI) structures—were initially designed and optimized using the RSoft environment [14]. The complete PIC is illustrated in Figure 2, and the operating principle of this architecture is described in the following subsection.

### 2.2. Operating Principle

The operation of the proposed PIC is graphically represented in Figure 2. Optical excitation is delivered via a single-mode optical fiber (SMF-28), coupling the optical field into the main structure through an optimized grating coupler. The coupled TE_0_ mode is transferred to a single-mode waveguide with an inverted planar taper, ensuring a low-loss transition by matching modal field profiles.

The optical signal is split equally by a 1 × 2 MMI, generating two coherent beams of identical amplitude. These beams are then fed into a 2 × 4 MMI, operating as a balanced optical power distributor and producing four nearly identical optical signals in amplitude and phase. Consequently, the optical power is uniformly distributed across the four parallel waveguide branches. The four optical branches are sent through the first electro-optic phase-shifting section, which enables independent modulation of each channel by applying an external DC bias at the control electrodes. These phase shifts form the initial stage of the reconfigurable routing mechanism and influence the interference conditions in the subsequent 4 × 4 MMI coupler.

Next, the four coherent optical signals are combined based on the self-imaging principle, with output signal redistribution determined by the induced phase distribution in the first electro-optic section. These optical outputs then go through a second electro-optic phase-shifting section, providing further phase control before reaching the final output routing 4 × 4 MMI.

The output routing 4 × 4 MMI is designed to ensure the coherent recombination of optical fields. This configuration features approximately double the propagation length compared to the preceding MMI, allowing deterministic routing of optical power to any output port and enabling arbitrary selection of the desired output channel, while suppressing unwanted outputs through specific phase shifts applied in both electro-optic sections.

### 2.3. Self-Imaging in Multimode Interference Couplers

The optical routing functionality of the proposed PIC uses MMI couplers based on the self-imaging principle. This principle imposes periodic imaging of the input optical field along the propagation direction due to constructive interference among guided modes. It offers a robust and compact method for creating power splitters, combiners, and optical routers with high fabrication tolerance and broad operational bandwidth.

The self-imaging effect arises from the superposition of guided modes in the multimode section, where each mode has a different propagation constant ($\beta_{m}$), resulting in a periodic redistribution of the optical field. The characteristic beat length between the two lowest-order modes ($\beta_{0}$ and $\beta_{1}$) is defined as:

(1) $$L_{\pi }\;=\;\frac{\pi }{\beta_{0}\;-\;\beta_{1}}.$$

The presence of single/multiple self-images depends on the relative phase accumulated by the propagating modes, which can be achieved at specific locations at multiples of the beat length, based on the chosen self-imaging configuration.

In the proposed architecture, we designed 1 × 2, 2 × 4, and 4 × 4 MMI couplers, and the initial dimensional estimates of the multimode sections were extracted directly from self-imaging theory. These preliminary dimensions were subsequently optimized using BeamPROP simulations to enhance power uniformity at the output ports and reduce excess and insertion losses, while meeting specific interference criteria essential for arbitrary optical routing. This optimization process also considered practical factors that the analytical model overlooks, such as the accurate refractive index profile and waveguide transition constraints.

The use of MMI couplers offers several advantages over directional couplers, including reduced sensitivity to dimensional variations, relaxed fabrication tolerances, compact footprint, and inherent broadband operation. These characteristics make MMIs particularly suitable for large-scale photonic integrated circuits requiring cascading optical routing stages, which are extremely useful in the proposed ultra-fast 1 × 4 optical switch.

The theoretical foundation of self-imaging in multimode waveguides has been thoroughly examined and is well-documented in the existing literature [7,8,9]. Detailed analyses concerning image formation conditions and MMI design methodologies are readily available [15]. In the present work, these fundamental principles have been used as the guiding rules for the numerical optimization and integration of the MMI couplers within the proposed PIC.

### 2.4. Numerical Modeling

The proposed PIC was designed and optimized using the software platform RSoft Photonic Device Tools from Keysight. Several numerical solvers were employed based on the specific physical phenomena under investigation. The BeamPROP module, which utilizes the Beam Propagation Method, was used to simulate and optimize passive optical components such as the MMI structures and, together with the Multiphysics module and the Mode Solver, was utilized to model the electro-optic phase-shifting regions, incorporating the effective Pockels coefficients reported in the literature.

For scenarios requiring full-wave electromagnetic analysis to validate the optical response of selected structures, such as the grating couplers and planar taper/inverted taper waveguides simulations, the FullWAVE finite-difference time-domain (FDTD) solver was employed. The modal indices of the guided modes were calculated using the RSoft Mode Solver. All simulations were executed at an operating wavelength of 1550 nm, considering propagation of the fundamental TE_0_ guided mode.

The complete PIC was optimized through successive simulations of the individual building blocks, followed by full device validation where all possible switching states were considered. The numerical results obtained for each optical element and for the complete switching architecture are presented and discussed in the Results section.

### 2.5. Electro-Optic Phase-Shift

The phase-shifting regions serve as the active elements of the proposed PIC and are responsible for controlling the interference conditions regulating the output port selection. However, a-Si does not exhibit an intrinsic linear electro-optic response when subjected to an external DC bias (Pockels effect), since it is a centrosymmetric material. To overcome this limitation, a layer of 100 nm SiN is assumed to be deposited over the a-Si core waveguide, inducing stress at the a-Si/SiN interface and promoting local symmetry breaking and the generation of an effective second-order nonlinear susceptibility ($\chi_{eff}^{2}$), as reported in the literature [16,17,18], when the external DC bias is applied in the racetrack waveguide regions.

The electro-optic behavior was modeled using the linear Pockels approximation, in which the refractive index variation is proportional to the applied electric field. The corresponding index variation is expressed as:

(2) $$∆n\;=\;-\frac{1}{2}n^{3}r_{eff}E$$

where ∆n is the change in the modal index of the guided mode, $r_{eff}$ is the effective electro-optic coefficient, and *E* is the applied DC electric field.

The induced phase-shift accumulated over an interaction length L is given by:

(3) $$∆\phi =\;\frac{2\pi }{\lambda }∆n_{eff}L$$

where ∆ϕ is the phase-shift, λ is the operating wavelength, ${∆n}_{eff}$ is the change in the modal index, and L is the length on which the DC bias is applied. Consequently, the interaction length to induce a π phase-shift is:

(4) $$L_{\pi }=\frac{\lambda }{2\left|∆n_{eff}\right|}$$

The electro-optic phase shifters were implemented in RSoft Multiphysics, where the refractive index variation was calculated from the applied electric field using the worst-case scenario of the effective electro-optic coefficients reported in the literature [12,13] for a-Si/SiN structures under DC bias. The selected coefficient was adopted exclusively for numerical modeling purposes and does not represent an experimental characterization performed within the scope of this work.

The effectiveness of the assumed electro-optic model in enabling arbitrary optical routing is demonstrated by the numerical results presented in the next section.

## 3. Results

As mentioned in Materials and Methods, our device consists of five identical fiber-grating couplers ended by taper waveguides, four MMI structures with different lengths, and eight racetrack waveguides, all connected through single-mode waveguides. In this section, we will be reporting the results obtained by simulation of the designed structures. This process is divided into three main simulations, since simulating the whole structure is next to impossible given the design complexity and the step size requirements of the smallest features (i.e., the holes in the metamaterial regions). Nevertheless, real world compliance is assured throughout the process by using tools that implement the required physics for each simulation task. Moreover, this device has been idealized to operate within the C-band of optical communications, hence the operating wavelength of 1550 nm has been selected for all simulations. Figure 2 presents the graphical description of the complete photonic circuit from the input to the optical fibers output.

### 3.1. The Grating Coupler and Planar Taper Waveguides

The five grating couplers in our device are all identical and can operate either as an input or output coupler. Thus, it will suffice to report the obtained results for one of the structures to assess the operating functionality and performance of all grating couplers, if we consider the structure operating both as output (grating–fiber) or input (fiber–grating) couplers. Since these couplers are establishing an optical link between different cross-section elements, optical fiber, and single-mode waveguides, a taper waveguide is mandatory to resolve the cross-section mismatch. In our device, this has been accomplished through planar taper waveguides, which can adapt the different cross-section waveguides within a much shorter length, when compared to the traditional linear/parabolic/spline taper waveguides.

#### 3.1.1. Grating–Fiber and Fiber–Grating Couplers

**Figure 3:**
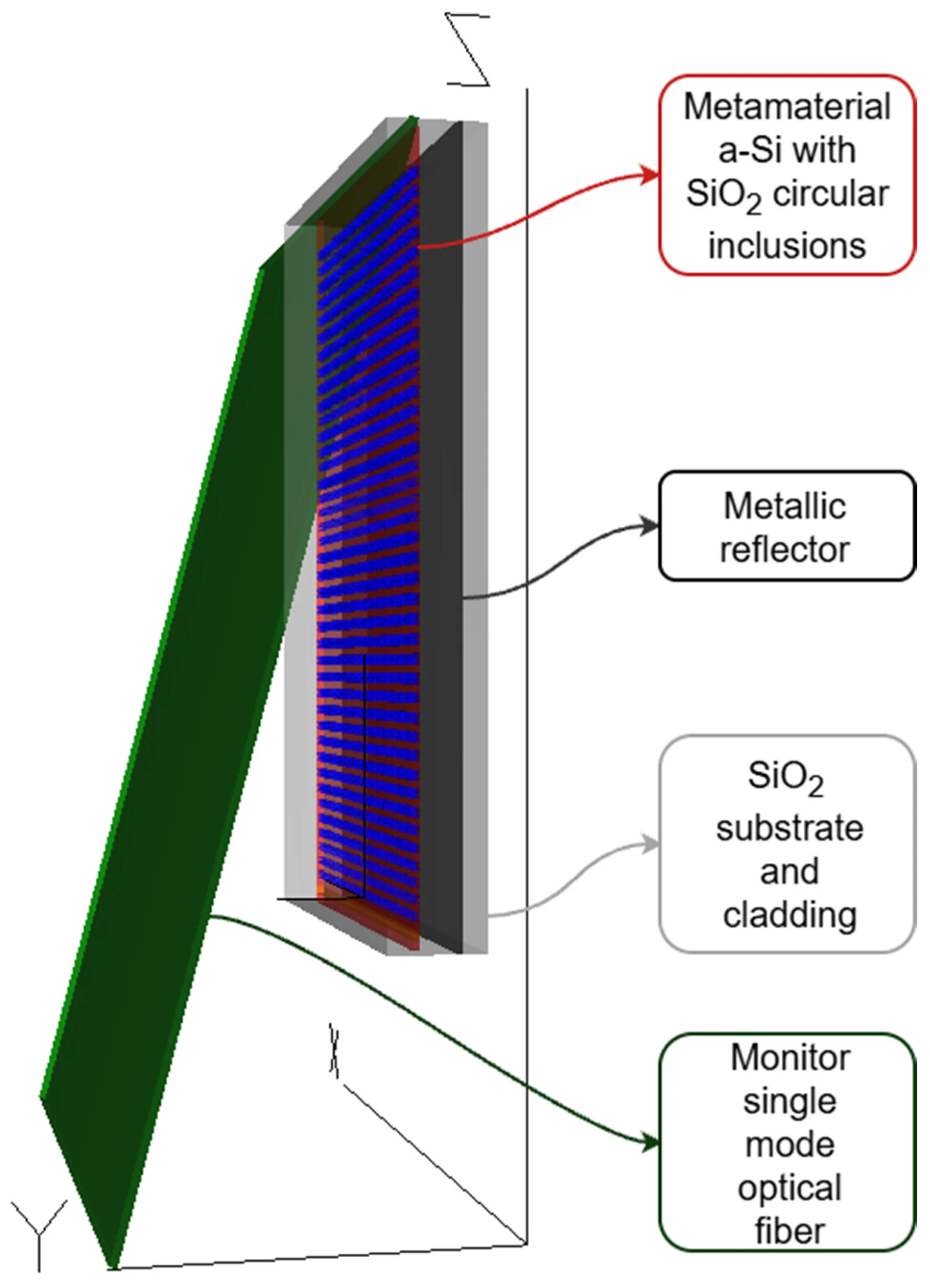
3D representation of the grating coupler consisting of a metamaterial structure formed by an a-Si core waveguide with circular inclusions, 14.6 µm wide and embedded in SiO_2_. Beneath the metamaterial structure there is a metallic film 100 nm thick and, above at an angle, a monitor to represent the single-mode optical fiber SMF28 (10.4 um diameter).

The grating coupler presented in Figure 3 has been developed to accomplish its functionality through the inclusion of a metamaterial structure, which has been engineered with the assistance of the genetic algorithm included in the scanning/optimization tool of our computational platform [14].

**Figure 4:**
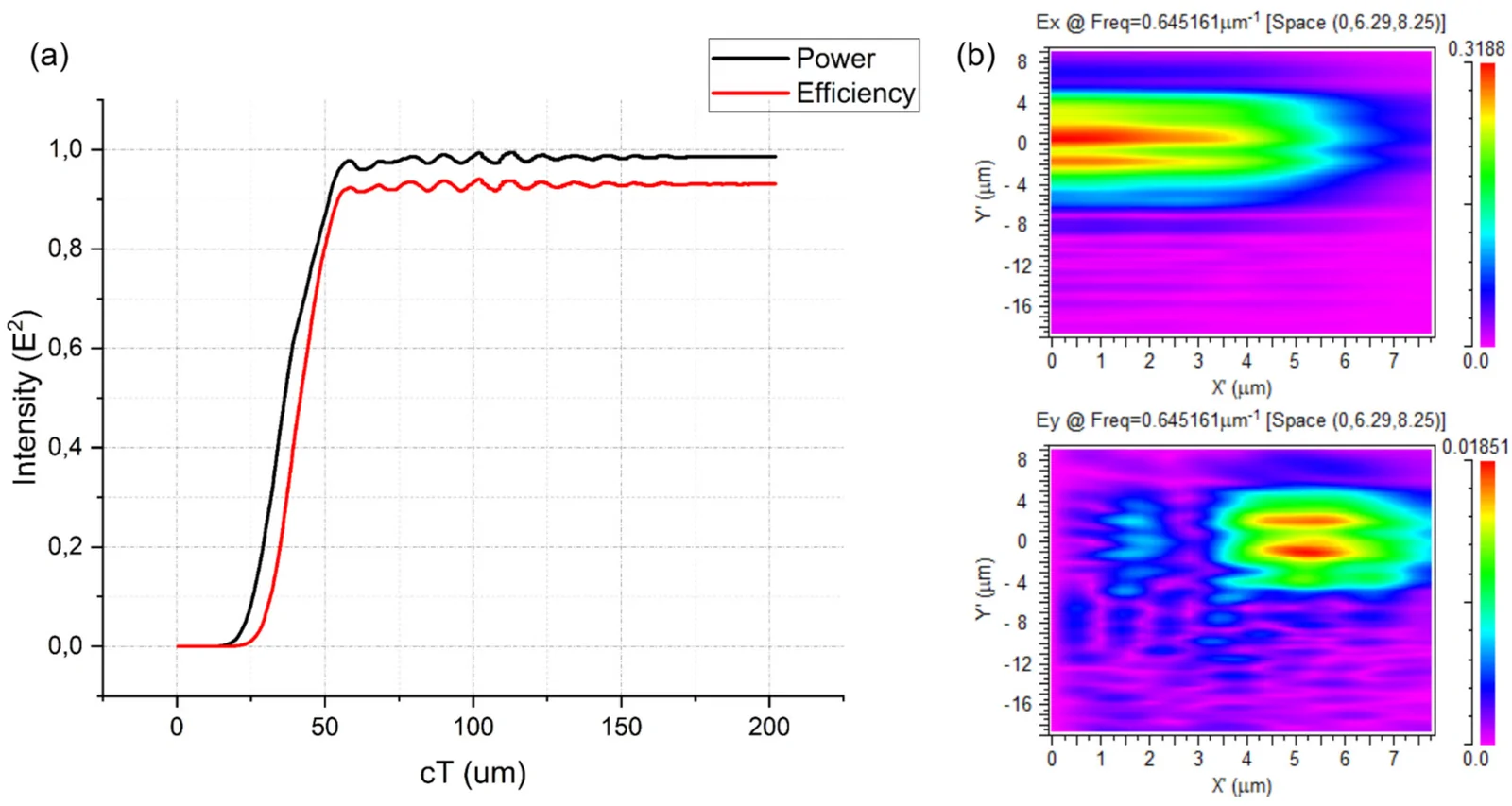
3D FDTD simulation results: (**a**) Power transfer (98.61%) and coupling efficiency (93.12%) into the TE0 mode of the core waveguide; and (**b**) Ex and Ey field profiles (only half profile is shown because we exploited symmetry in simulation along X- and Y-axes).

This structure was then simulated at the 1550 nm operating wavelength with the FDTD tool of the computational platform to assess the structure’s coupling efficiency into the fundamental mode of the optical fiber. The simulation considered the TE_0_ mode of the 14.6 µm wide a-Si core waveguide, propagating along +Z in Figure 3, with a 10 nm step size to assure discretization of the circular inclusions and taking advantage of the structure’s symmetry along X- and Y-axes to optimize the simulation time. Figure 4 shows the results obtained for both the power amplitude transfer and coupling efficiency to the mode of the optical fiber. With this configuration we were able to obtain a coupling efficiency of 93.12% and a power transfer of 98.61%, from the grating structure to the SMF28 optical fiber.

**Figure 5:**
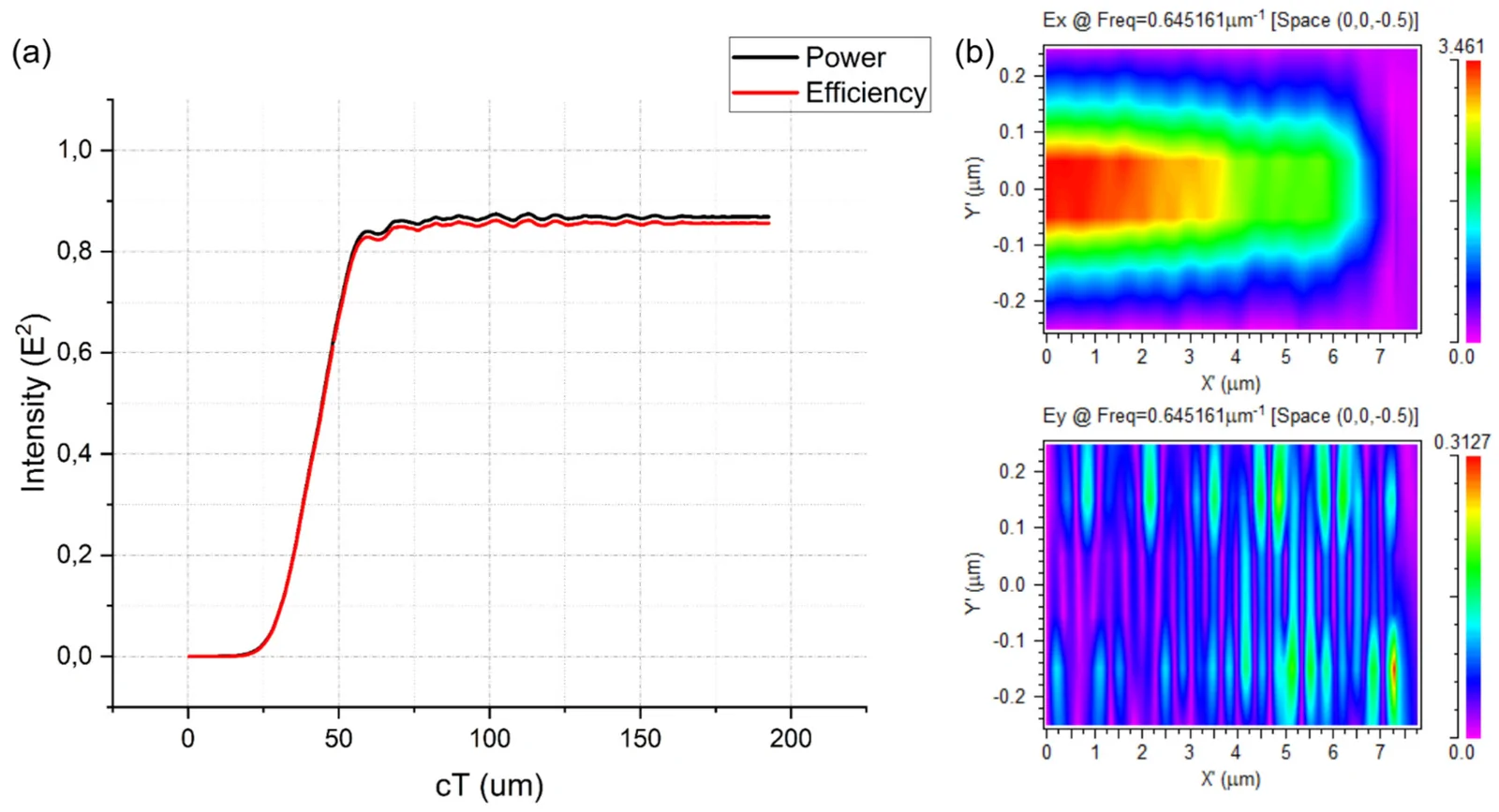
3D FDTD simulation results: (**a**) Power transfer (86.86%) and coupling efficiency (85.62%) into the TE_0_ mode of the core waveguide; and (**b**) Ex and Ey field profiles (only half profile is shown because we exploited symmetry in simulation along X- and Y-axes).

Next, we considered the same geometric configuration propagating in the opposite direction to assess the structure’s efficiency when the input beam is directed at the grating coupler and coming from the SMF28 optical fiber. The results obtained are presented in Figure 5, and in this case, we achieved 86.86% power transfer into the core waveguide and 85.62% coupling efficiency to the TE_0_ mode. If comparing power transfer and coupling efficiency between previous simulations results, one can observe a slight decrease in the latter. Although effects like sidewall roughness, local etch non-uniformity, and process-dependent size variations severely impact performance in photonic structures [19], this decrease in coupling efficiency is explained by a foundry constraint related to the vertical and lateral etch rates of a-Si, and pertains specifically to the circular features present in the grating couplers and taper waveguides:

- Reactive ion etching, although highly precise, uniform and selective, does not produce vertically perfect walls of the circular inclusions in a-Si;
- Foundry specifications were assumed to be 25 nm/min and 2.5 nm/min/side for vertical and lateral etch rates, respectively, leading to a sidewall angle of ~83.4° for the circular inclusions in a-Si;
- To compensate for the sidewall angle, all circular inclusions have been biased in the design by 7°; this has favored the simulation coupling efficiency when propagating from the grating coupler to the SMF28 optical fiber, but introduces a slight decrease when propagation is in the opposite direction (i.e., from the SMF28 optical fiber to the grating coupler).

#### 3.1.2. The Planar Taper Waveguide

This work builds on previous research on planar taper waveguides operating in both the visible [20,21,22] and, more specifically, the near-infrared range of the electromagnetic (EM) spectrum [23]. The results presented in this section characterize the taper as an individual functional building block and are therefore not intended to provide an end-to-end insertion loss estimate for the complete PIC.

**Figure 6:**
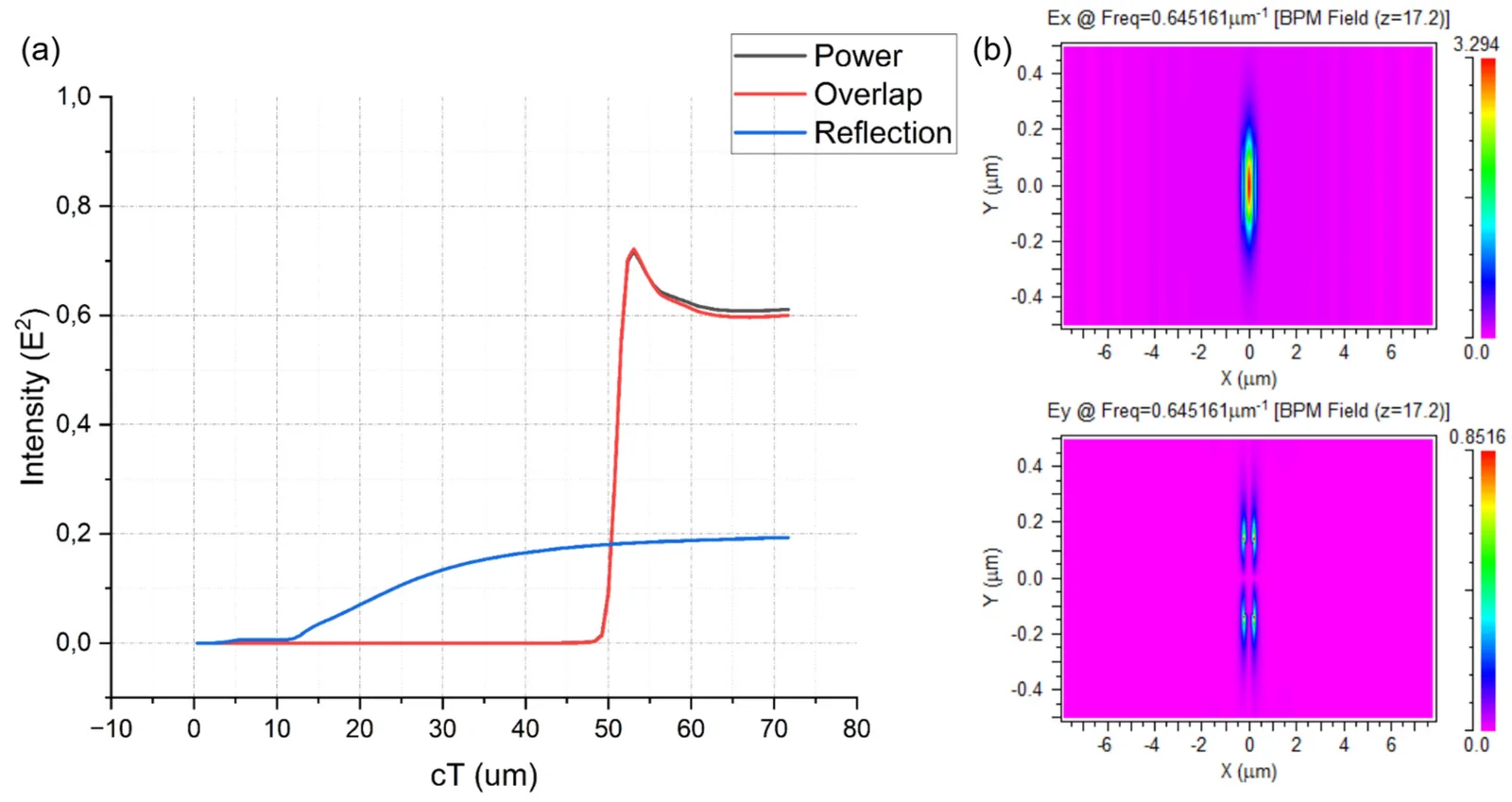
(**a**) Planar inverted taper efficiency, power transfer and reflection; (**b**) the modal profiles for both Ex and Ey fields.

Since the etching process is the same as before, the taper waveguides simulations accounted for the same constraints as the grating coupler. Hence, we expect obtaining different efficiency results when the planar taper waveguide operates as a grating coupler to single-mode waveguide adapter (inverted taper operation), or when it operates in the opposite direction (single-mode waveguide to grating coupler adapter–taper operation). Figure 6 presents the results obtained when operating as an inverted taper, where significant reflections (~20%) and efficiency or overlap integral (~60%) may be observed. The modal profiles presented have been acquired by a spatial monitor at the end of the simulation space and show good agreement with the spatial modal profile of the calculated single mode of the waveguide, along with scattered power outside of the waveguide boundaries.

**Figure 7:**
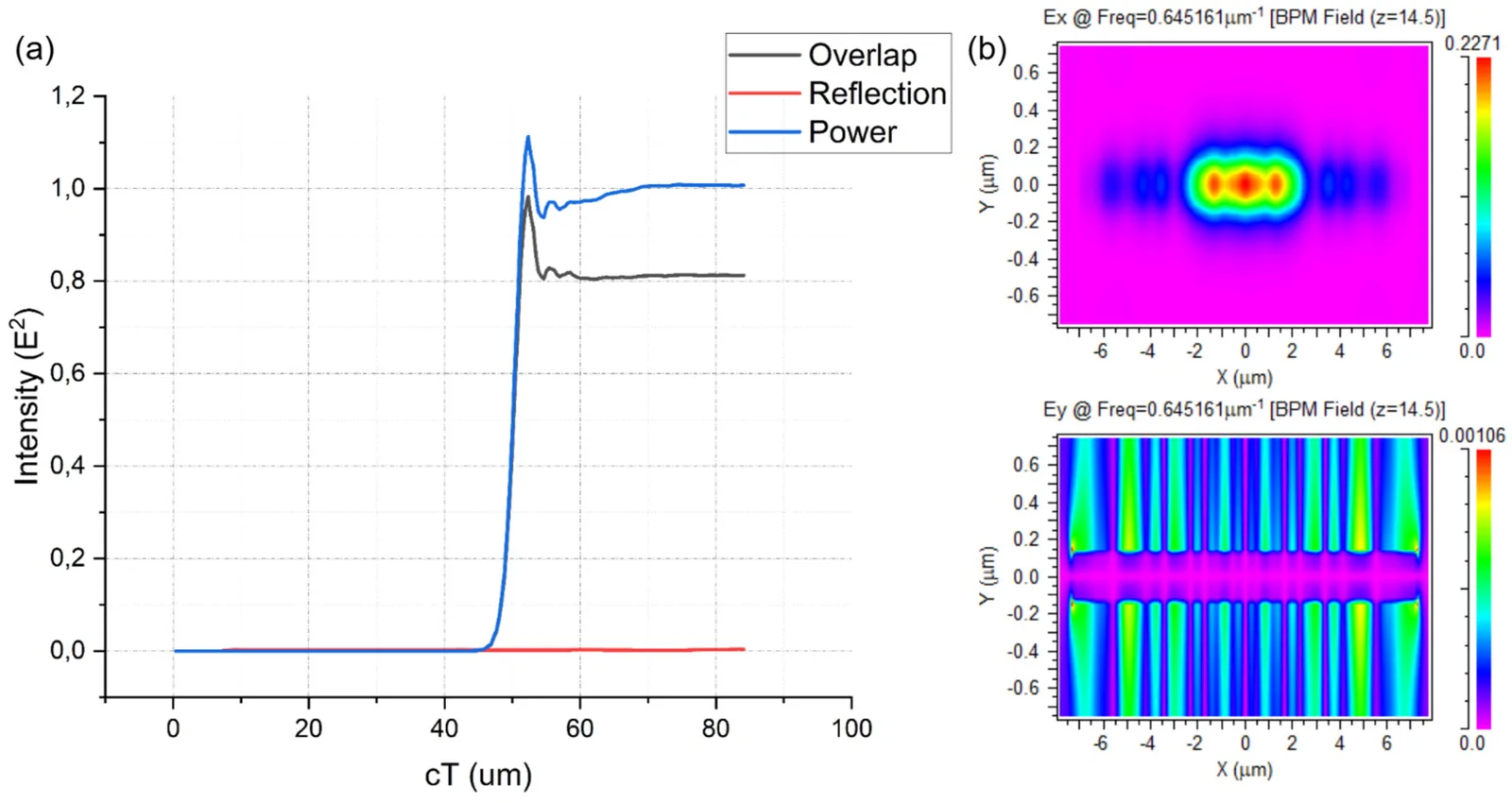
(**a**) Planar taper efficiency, power transfer and reflection; (**b**) the modal profiles for both Ex and Ey fields.

When operating in the reverse direction, the planar taper waveguide presented better results than the ones shown in Figure 6. Figure 7 shows the results obtained when the structure is operated as a taper waveguide. Here, we observe an efficiency of ~80%, power transfer of ~100% and an insignificant reflection level. One may hypothesize that the reflected power verified in Figure 6 may have been transferred to forward propagation, being added essentially to the observed efficiency (or overlap integral) in this case.

#### 3.1.3. The Phase-Shift Regions (Racetrack Waveguides)

The operating principle of the device considers implementing the Pockels effect to impact, with the required phase-shift, the mode propagating within the single-mode waveguides and assure arbitrary selection of the output optical fiber propagating the EM beam. Since this device is being fabricated in a SOI-compliant platform using a-Si as the core material, second-order non-linearity is practically absent in a-Si due to their structural centrosymmetricity. However, there have been reports in the literature [12,13] confirming the experimental observation of an effective second-order non-linearity (claimed to be Kerr related) that, although modest, is unequivocally present when the centrosymmetricity of Si based structures is broken.

**Figure 8:**
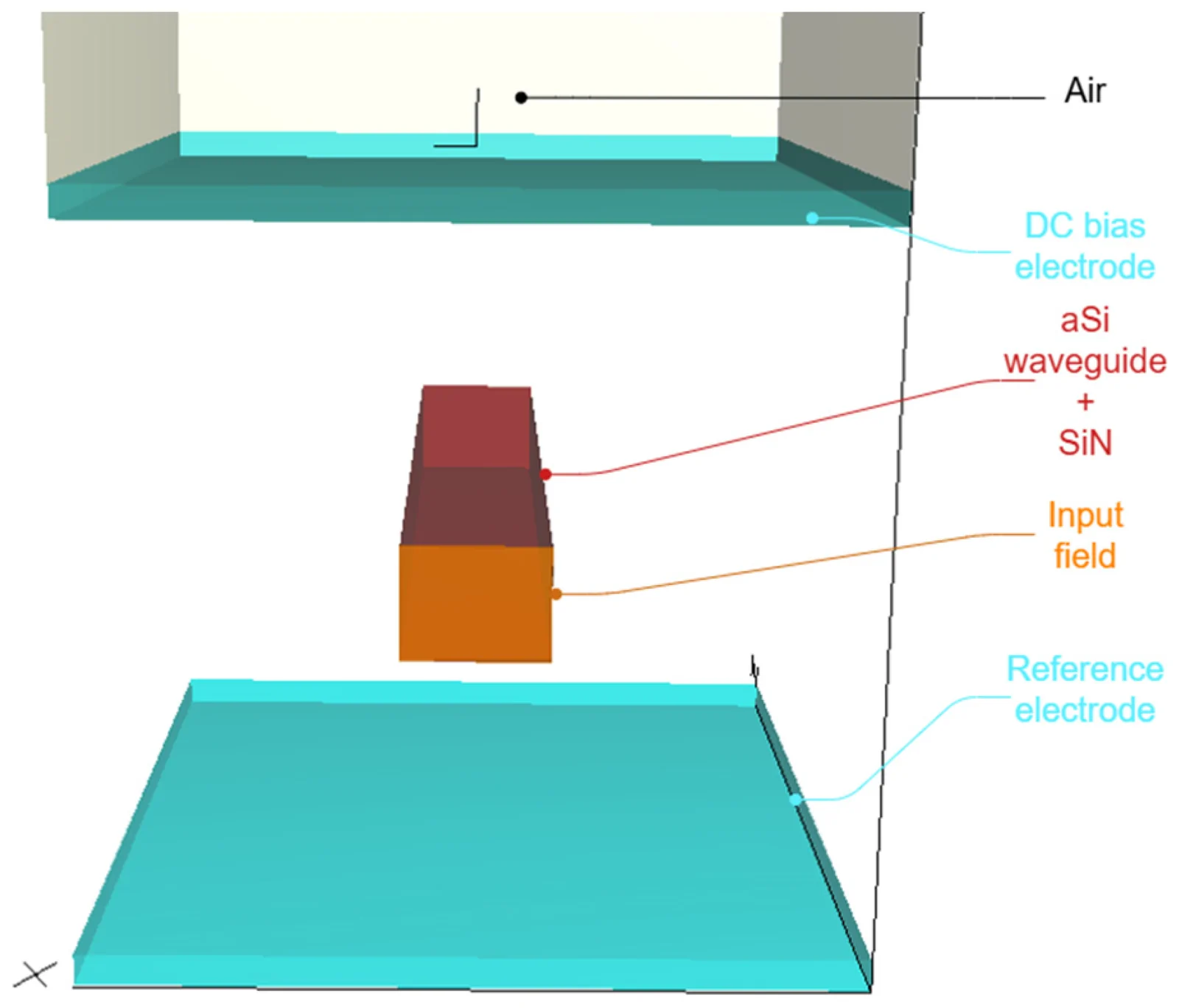
Simulated workspace considering the waveguide embedded in silicon dioxide and 2 electrodes (bottom light blue electrode is ground plane and DC electric potential is applied on top light blue electrode); air fills the region above the top light blue electrode.

The RSoft electro-optic model requires an effective linear electro-optic coefficient $r_{eff}$, as described in Equation (2), relating the local refractive index perturbation to the generated electric field. Since the software does not directly model strain-induced second-order susceptibility, the lowest experimentally reported effective electro-optic coefficient for strained Si (0.5 pmV^−1^) was adopted [12] as phenomenological parameter to reproduce the measured electro-optic response. The simulation workspace is presented in Figure 8 and represents an a-Si waveguide 450 nm wide and 250 nm high, topped by a SiN layer 450 × 100 nm (multilayer structure in RSoft) and embedded in SiO_2_. There are also two electrodes (light blue structures) where the DC bias is applied, and the region above the top electrode is air.

Since the phase-shift induced by an applied DC electric potential is described by Equation (3), the conducted strategy to determine the required waveguide length to impart a π phase-shift to the propagating mode consisted of calculating the modal indices of the modes with and without the applied DC bias, obtaining the difference between the two modal indices, and directly substitute in Equation (4) to retrieve length $L_{\pi }$.

**Figure 9:**
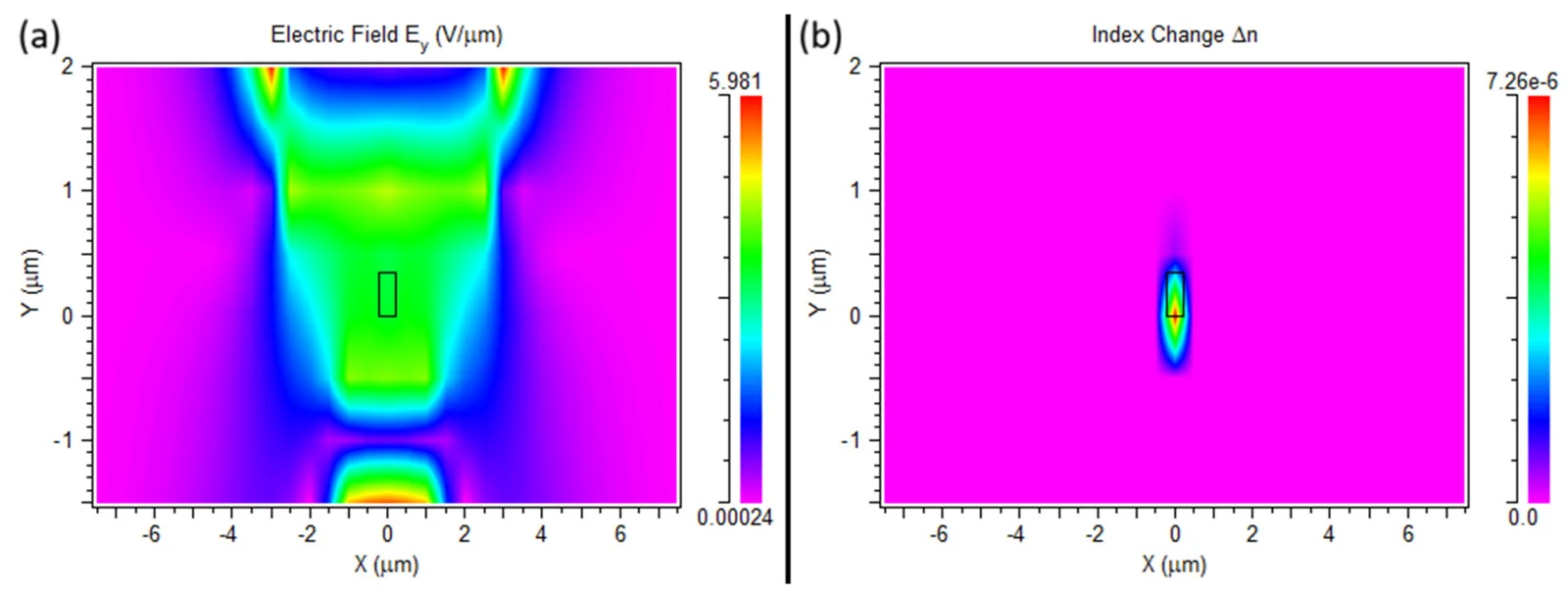
In both figures, the black rectangle represents the dimensional limits of the waveguide; (**a**) the resulting electric field in the XY plane; and (**b**) the refractive index change induced in the core waveguide by the generated electric field.

**Figure 10:**
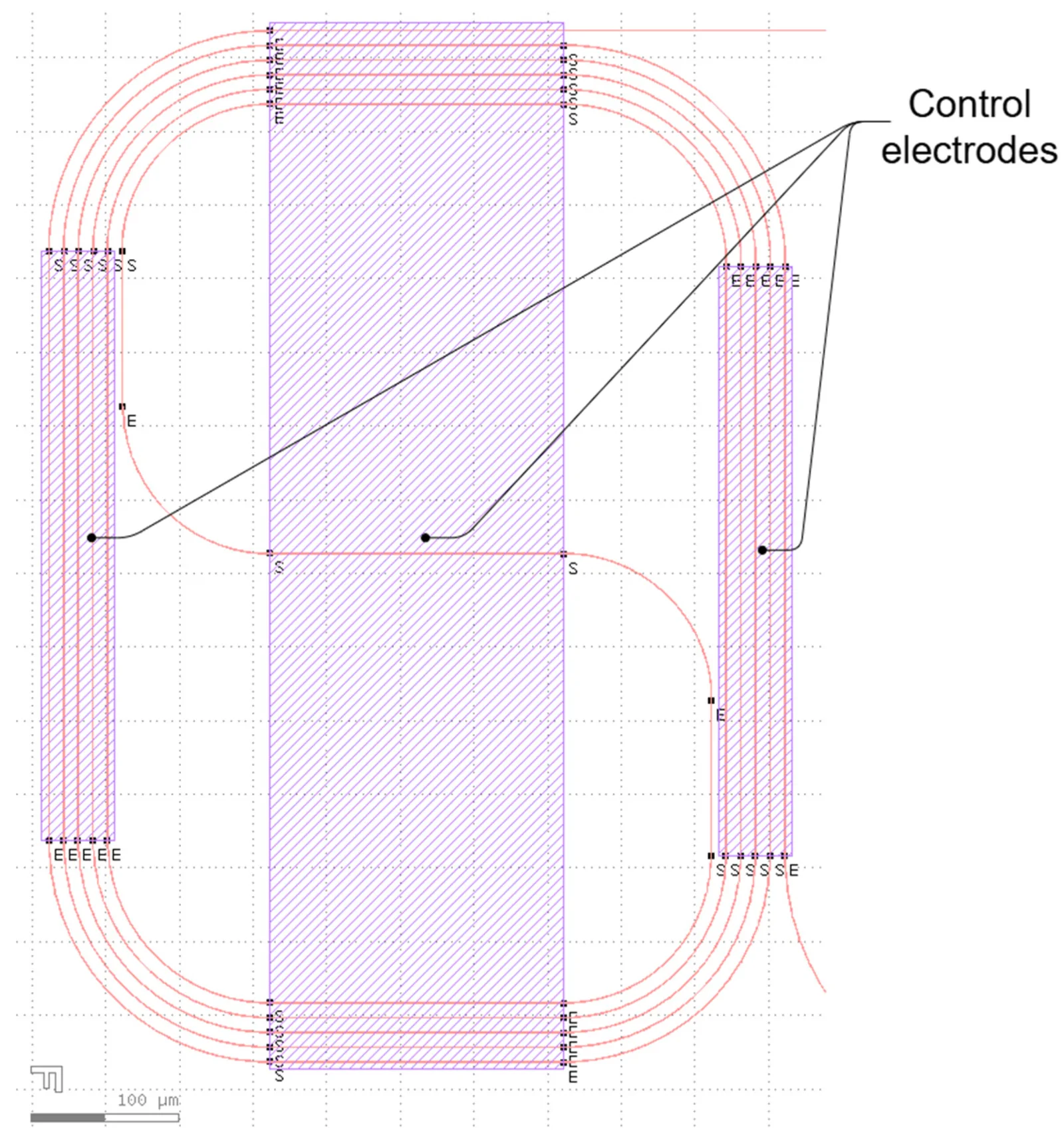
Racetrack waveguide with control electrodes and design scale.

Figure 9 shows the resulting electric field within the structure when considering an electric potential of 10 V applied to the electrodes and the corresponding changes in the refractive index:

Next, we evaluated the change in the refraction index, the change in the modal index of the propagating mode, and the length required to achieve a phase-shift of π ($L_{\pi }$ using Equation (4)), when the applied electric potential is 10, 15 and 20 V. Table 1 presents the obtained results:

Taking into consideration the results obtained, we designed the phase-shift structure consisting of a racetrack waveguide constrained by the $L_{\pi }$ values in Table 1. The whole control region consists of eight racetrack waveguides, distributed into two groups of four as depicted in Figure 2. Each racetrack waveguide is located between two MMI structures, connecting the output of the first to the input of the last MMI. Figure 10 shows a representative example of the designed structures, together with the control electrodes (scale at the bottom left).

Since the region covered by the electrodes in each racetrack waveguide spans to ~12.4 mm and the worst-case scenario for $L_{\pi }$ in Table 1 requires ~14.3 mm, we will have to apply a DC electric potential between 10 and 15 V when in operation and the phase control demands for π phase-shift.

#### 3.1.4. The Multimode Interference Structures

Since the ultimate intent of this work is the arbitrary selection of one in four output ports, we have designed a set of four cascaded MMI elements that are able to select the intended output port, by means of introducing phase shifts at specific locations along the optical path. The developed strategy is based in the work of Linh et al. [24], where the mathematical approach is described and a mode-selective switch has been designed and demonstrated through simulation. Our design performs as a 1 × 4 switch, thus connecting the input port to one of the four possible port outputs.

**Figure 11:**
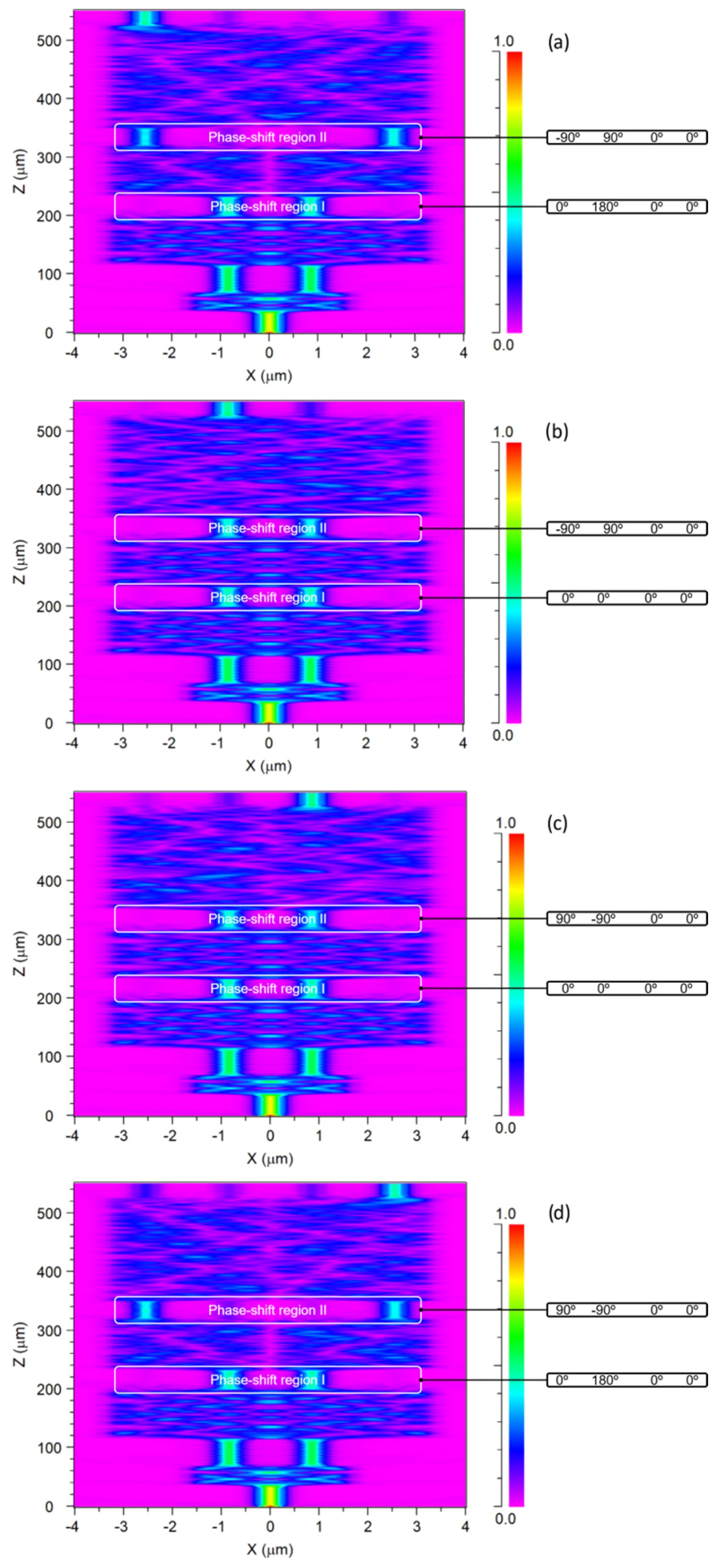
Output selection and phase-shift configurations at the electro-optic regions, illustrating a qualitative view of the routing function (not a quantitative end-to-end characterization). Input field is at the bottom and output fields at the top; (**a**) left output is selected; (**b**) left-center output is selected; (**c**) right-center output is selected; and (**d**) right output is selected.

The idealized structure is depicted in Figure 2 and consists initially of a 1 × 2 MMI, necessary to provide two spatially independent outputs for the next element, follows a 2 × 4 MMI, whose outputs feed the first electro-optical region consisting of four racetrack waveguides, capable of inducing a phase-shift in their propagating mode and which are then connected to the next element. The next element is a 4 × 4 MMI, that propagates the incoming EM fields from previous racetrack waveguides, delivers the output to the second electro-optic region containing four racetrack waveguides with phase-shift capabilities and connected to the next and last element of the MMI chain. The last MMI element simply propagates the incoming EM fields and, given the phase difference present in two of the input ports, the output port is selected. To facilitate visualization of the routing mechanism, the dynamic operation of the proposed architecture is depicted in Figure 11 and provided as Supplementary Video S1.

## 4. Discussion

The present numerical study focuses on demonstrating the reconfigurability of the cascaded MMI architecture and validating its constituent functional blocks. Consequently, global performance metrics requiring end-to-end modeling, such as total insertion loss, aggregate crosstalk and extinction ratio, are not derived from the component-level simulations.

### 4.1. Sensitivity Analysis of the Electro-Optic Phase Shifter

**Figure 12:**
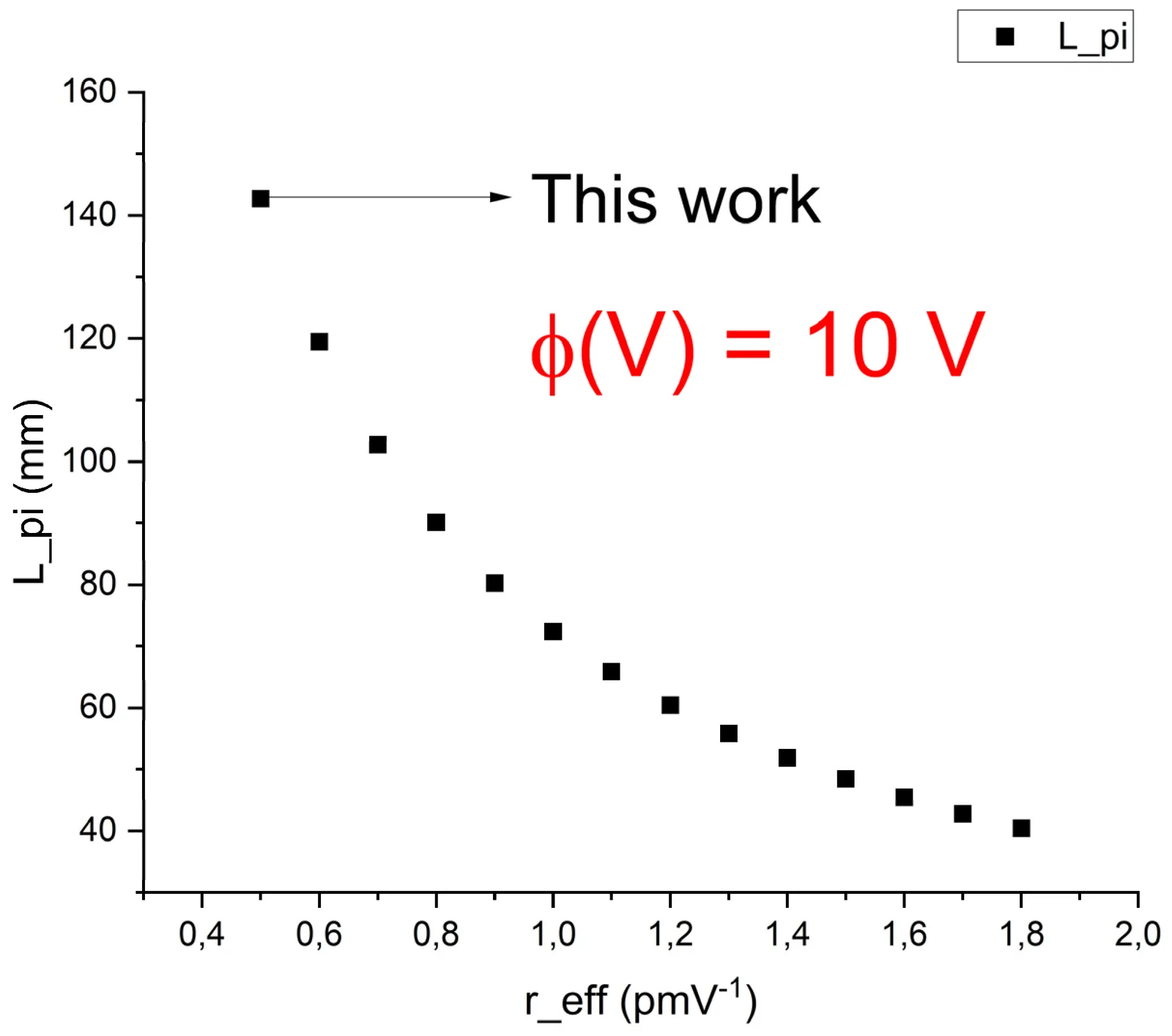
Sensitivity of *L*_π_ with the effective electro-optic coefficient *r*_eff_.

Although Table 1 already illustrates the dependence of the induced effective index change and the corresponding phase-shift length on the applied voltage, an additional sensitivity analysis was performed to assess the influence of the uncertainty associated with the effective electro-optic coefficient reported in the literature [12,13]. Figure 12 shows the calculated variation of $L_{\pi }$ as a function of $r_{eff}$ for a fixed driving voltage of 10 V. As expected from the linear Pockels model, $L_{\pi }$ decreases approximately inversely with $r_{eff}$, while the operating principle of the proposed architecture remains unchanged.

Regarding the spectral response of the cascaded MMI structures and its impact in the overall performance of the device, although the individual MMI couplers exhibit broadband behavior, the overall spectral response of the complete architecture is governed by the cumulative phase relationships established across the cascaded interference stages. Owing to the presence of two independently controlled electro-optic phase-shifting sections, the proposed architecture offers the possibility of compensating wavelength-dependent phase variations through electrical tuning. A systematic investigation of the achievable spectral operating range and adaptive phase compensation will be the subject of future work.

### 4.2. GDSII Layout and Fabrication Readiness

The simulation results detailed in the preceding section affirm that the proposed architecture successfully satisfies all design requirements for an ultra-fast 1 × 4 all-optical switch. These requirements encompass efficient optical coupling, low-loss waveguide transitions, effective electro-optic phase control, and deterministic routing to any output port via cascaded MMI couplers. Collectively, these findings provide compelling evidence of the feasibility of the proposed device, facilitating its progression from numerical validation to experimental implementation. Accordingly, a fabrication proposal was submitted to INFRACHIP, a European-distributed research infrastructure that offers competitive access to advanced semiconductor manufacturing facilities. This proposal received approval, and the complete layout of the device was subsequently generated and submitted for fabrication.

Since preceding optical elements have been developed independently, the corresponding geometries were exported separately as Graphic Data System (GDS) files [25]. The GDS format represents the industry standard for semiconductor and photonic device fabrication, as it contains the geometrical and layer definitions required for mask generation.

Subsequently, the exported layouts were imported into KLayout [26], an open-source hierarchical editor extensively employed in the design and preparation of photonic integrated circuits. Within this environment, the individual photonic components were combined to construct the complete device layout. a-Si racetrack waveguides were then incorporated to interconnect the different sections of the optical switch and aligned with the MMI structures, thereby defining the optical propagation paths associated with the 1 × 4 switching operation.

Following the completion of the photonic layer, the metallization layers containing the electrical contacts were added onto the regions of the racetrack waveguides. These metallic electrodes enable the application of an external electric potential, which induces refractive-index variations through the effective Pockels effect, thereby allowing dynamic control of the optical phase within the device.

Next, and following foundry recommendations, the small circular holes present in the grating couplers and in the planar taper waveguides were switched to a negative mask configuration. This change enhances efficiency and accuracy in electron-beam lithography fabrication, as the circular electron beam can directly expose these features, unlike a positive mask, which requires more time and affects pattern fidelity.

Furthermore, polygons exported from RSoft platform had irregular vertex distributions and non-ideal geometries, impacting the device’s dimensional accuracy. To address this, a Python correction algorithm was developed using the gdstk library. It imports the GDS file, identifies candidate small circular structures based on geometric criteria like circularity and vertex count, and applies a least-squares circle-fitting method to optimize their shapes. The corrected polygons are then reconstructed with uniform vertex distribution, snapped to a 1 nm design grid, and an updated GDS file is generated.

**Figure 13:**
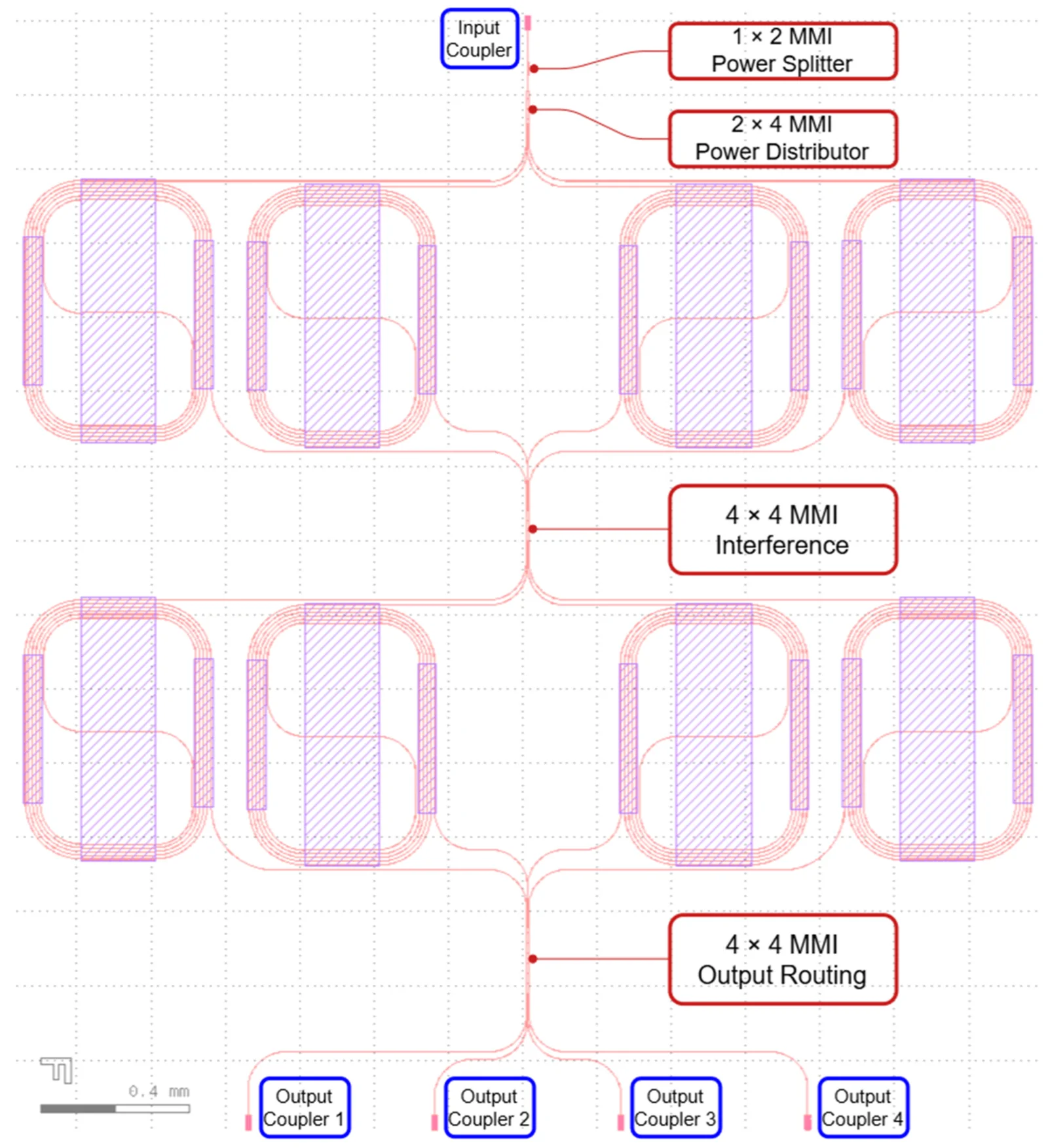
GDS file of the ultra-fast all-optical 1 × 4 switch which has been sent for fabrication.

The adoption of a hierarchical design strategy ensured precise control over the fabrication process and guaranteed the accurate alignment of the various structural elements, including the silicon waveguides, tapers, MMI structures, racetrack waveguides, and metallic electrodes. The resulting layout file therefore contains all the information required for the fabrication of the multilayer mask employed in the INFRACHIP manufacturing process and is presented in Figure 13.

The availability of fabricated prototypes will also enable experimental assessment of long-term electro-optic stability, including the influence of thermal fluctuations, charge trapping and electric-field screening under continuous DC bias, aspects that were intentionally excluded from the present numerical study.

## 5. Conclusions and Further Work

This work describes the design and numerical validation of a reconfigurable electro-optic PIC architecture using cascaded multimode interference couplers and electro-optic phase shifters on an amorphous silicon platform. The architecture accomplishes dynamic routing of optical signals across four output ports while remaining compatible with CMOS fabrication technologies.

Rather than optimizing individual photonic components, this study presents a comprehensive design methodology that combines passive optical elements, electro-optic phase control, and numerical multiphysics modeling into a unified, reconfigurable PIC architecture. The modular design approach offers a scalable framework for creating more complex programmable photonic circuits.

The maturity of this architecture is evidenced by the completion of a fabrication-ready GDSII layout, submitted through the INFRACHIP European access program. This marks a significant step toward experimental validation of the concept.

This architecture constitutes a practical step towards scalable, CMOS-compatible, and reconfigurable photonic integrated circuits capable of supporting future ultra-fast optical communication and signal processing systems.

Future efforts will include characterizing the fabricated device for insertion loss, crosstalk, extinction ratio, and switching speed, as well as expanding the architecture to larger switching matrices and more advanced programmable photonic circuits.

## Figures and Tables

**Table 1:** The results obtained for electric potentials of 10, 15 and 20 V.

ϕ (V)	∆n (Max)	$∆n_{eff}$	$L_{\pi }$ (mm)
10	7.26 × 10^−6^	5.429 × 10^−6^	142.75
15	1.09 × 10^−5^	8.143 × 10^−6^	95.17
20	1.45 × 10^−5^	1.086 × 10^−6^	71.36

## Data Availability

The original contributions presented in this study are included in the article/Supplementary Material. Further inquiries can be directed at the corresponding author.

## Supplementary Materials

The following supporting information can be downloaded at: https://www.mdpi.com/article/10.3390/mi17091035/s1, Video S1: Reconfigurable PIC.

## Abbreviations

The following abbreviations are used in this manuscript:

PIC	Photonic Integrated Circuit
CMOS	Complementary Metal-Oxide-Semiconductor
MMI	Multimode Interference
a-Si	Amorphous Silicon
SOI	Silicon-On-Insulator
SiN	Silicon rich Nitride
DC	Direct Current
TE	Transverse Electric
EM	Electromagnetic
GDS	Graphic Data System
FDTD	Finite-Difference Time-Domain
